# Reverse dorsoradial metacarpal artery flap for reconstruction of large finger skin defect: A case report

**DOI:** 10.1016/j.ijscr.2023.108610

**Published:** 2023-08-06

**Authors:** Dedy Chandra Hariyono, Affandi Wiramur, Amru Sungkar, Kristanto Yuli Yarso, Richard Philo

**Affiliations:** a Department of Surgery, Moewardi General Hospital, Jl. Kol. Sutarto No. 132, Surakarta, Indonesia; b Plastic Surgery Division, Deparment of Surgery, Moewardi General Hospital/Sebelas Maret University, Jl. Ir Sutami No. 36 a, Surakarta, Indonesia; c Sebelas Maret University, Jl. Ir Sutami No. 36 a, Surakarta, Indonesia; d Surgery Department, Oncology Division, Sebelas Maret University, Ir Sutami Street 46 ^th^ Solo Central Java, Indonesia

**Keywords:** Finger defects, Reconstruction, Reverse dorsoradial metacarpal artery flap

## Abstract

**Introduction and importance:**

Plastic reconstructive surgery for defects of hand still pose a challenge to plastic surgeons. The skin envelope of the hand is a complex structure that not only covers the underlying tissues but also includes particular functional and sensory components. The main principle of therapy is to provide adequate and long-lasting coverage to minimize surgery scars.

**Case presentation:**

A 63 years old man was referred to our hospital from another facility with a soft tissue defect on his right index finger at the level of the interphalangeal joint. The injury occurred 4 hours prior when he was injured by a sickle while working in a rice field. The defect was measured 2 × 4 cm and the base was visible, exposing the underlying fascia with minimal contamination. The patient underwent treatment with a reverse dorsoradial metacarpal artery flap, which was elevated from the thumb. After one month, he returned to the hospital with complains of pain and drainage from the donor site over the past few days. Debridement was performed and the area was repaired with local anesthesia. Patient didn't complains about the functional impairment.

**Clinical discussion:**

Compound defects of the hand need urgent surgical intervention and flap covering to prevent stiffness, improve the range of motion and facilitate early return to work. The reverse dorsoradial metacarpal artery flap is an excellent option for finger defects reconstruction.

**Conclusion:**

This study showed that the reverse dorsoradial metacarpal artery flap is a viable flap for reconstructing finger defects.

## Highlights

• Plastic reconstructive surgery for defects of hand still pose a challenge to plastic surgeons.
• Adequate coverage is necessary to replace missing skin and safeguard exposed structures.
• The reverse dorsoradial metacarpal artery flap is a excellent option for covering dorsal finger defects.

## 1 Introduction

The main principle of therapy for soft tissue abnormalities of hands is to provide adequate and long-lasting coverage to minimize surgery scars [1]. Various surgical procedures can be used, such as the use of a fasciocutaneous flap [2]. In 1990, Quaba and Davison introduced the dorsal metacarpal artery flap (DMCA) which uses a constant palmar-dorsal perforator presents in the digital web space. This technique became a popular choice for covering defects on the dorsal side of the fingers up to the PIP joint [3]. Another type of flap that widely used is the reverse dorsal metacarpal artery (RDMA) flap, which is raised from the dorsal of the hand and nourished by the dorsal metacarpal artery and/or the palmar arterial system through dorsopalmar anastomosis. These flaps are often used for covering defects on the dorsal side of the fingers [4]. The objective of this study was to evaluated the applications of reverse dorsoradial metacarpal flap for finger defect reconstruction. The case report adheres to SCARE criteria [5].

## 2 Case report

**Fig. 1:**
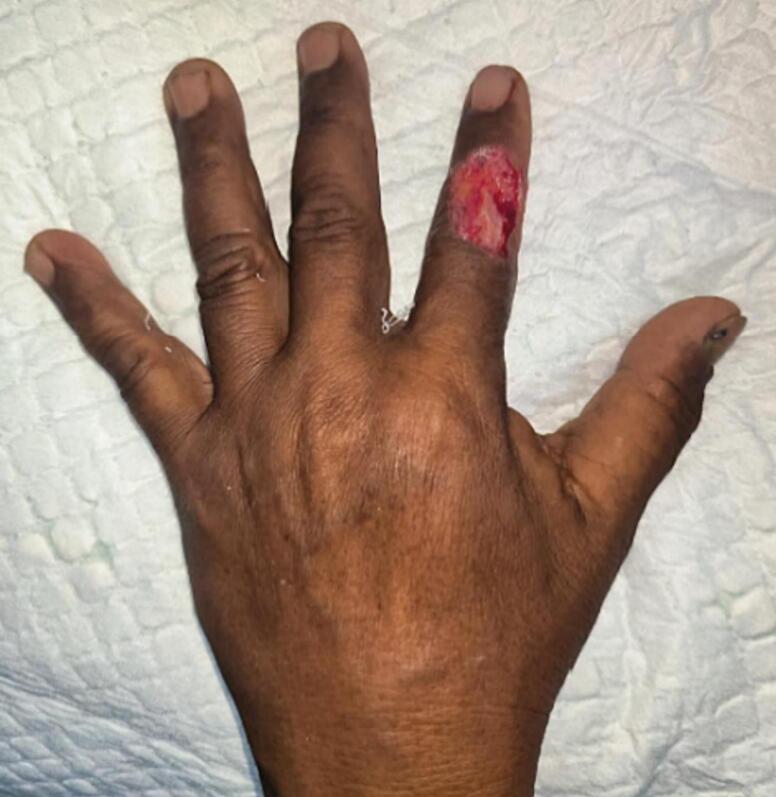
Defect at the interphalangeal joint of right index finger.

A 63-year-old man was referred to our hospital from another facility with a soft tissue defect on his right index finger at the level of the interphalangeal joint. The injury occurred 4 h prior when he was injured by a sickle while working in a rice field. The defect was measured 2 × 4 cm and the base was visible, exposing the underlying fascia with minimal contamination (Fig. 1). The patient underwent treatment with a reverse dorsoradial metacarpal artery flap, which was elevated from the thumb.

**Fig. 2:**
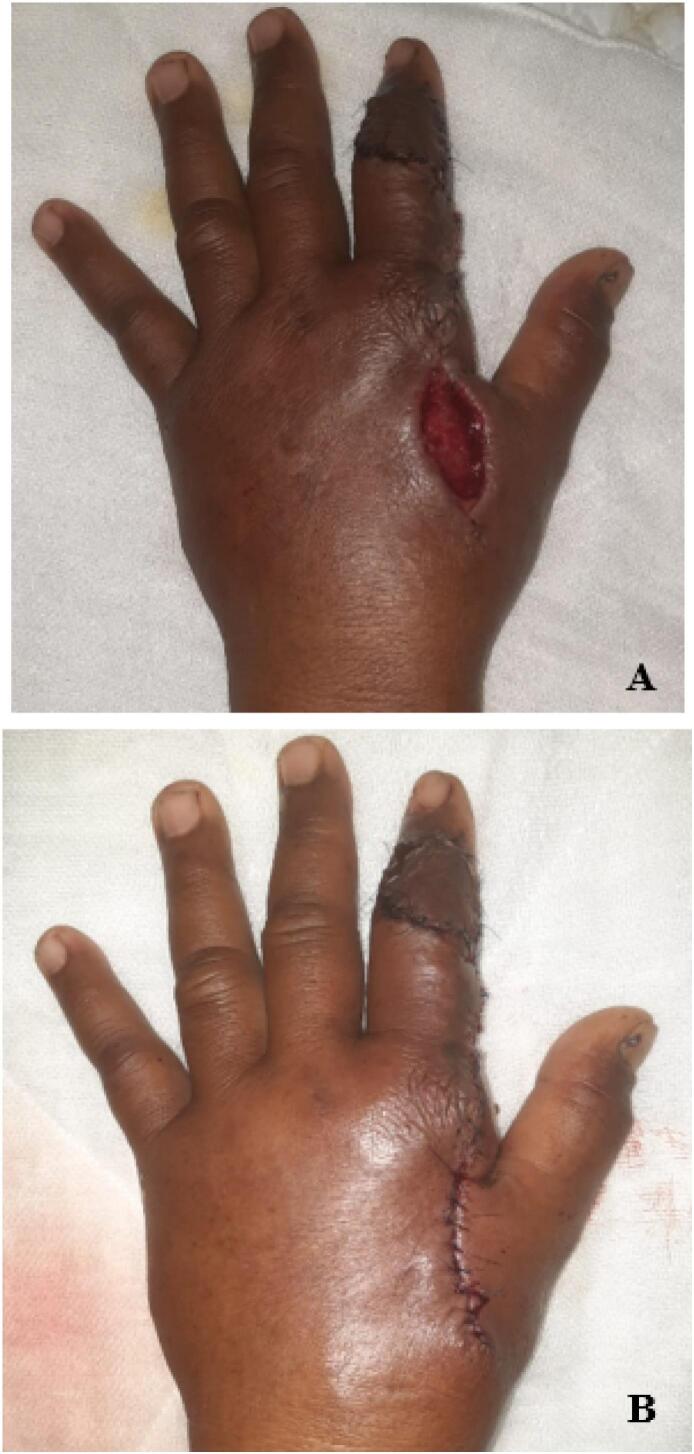
(A) Dehiscence wound after 1 month post operative. (B) Debridement and repaired using local anesthetic.

After one month, he returned to the hospital with complains of pain and drainage from the donor site over the past few days (Fig. 2a). Debridement was performed and the area was repaired with local anesthesia (Fig. 2b). Patient didn't complains about the functional impairment.

## 3 Operation technique

**Fig. 3:**
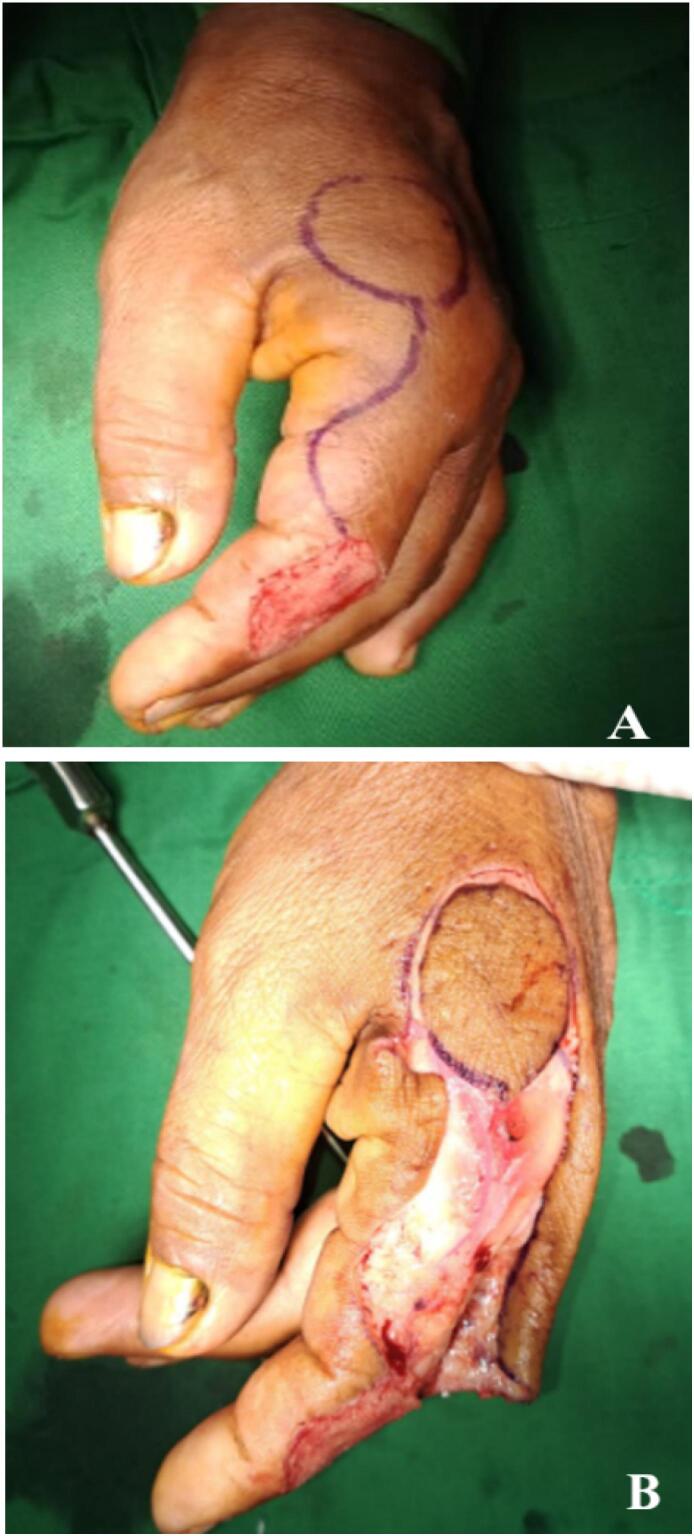
(A) Planning of reverse dorsoradial artery flap. (B) Elevated dorsoradial metacarpal flap.

**Fig. 4:**
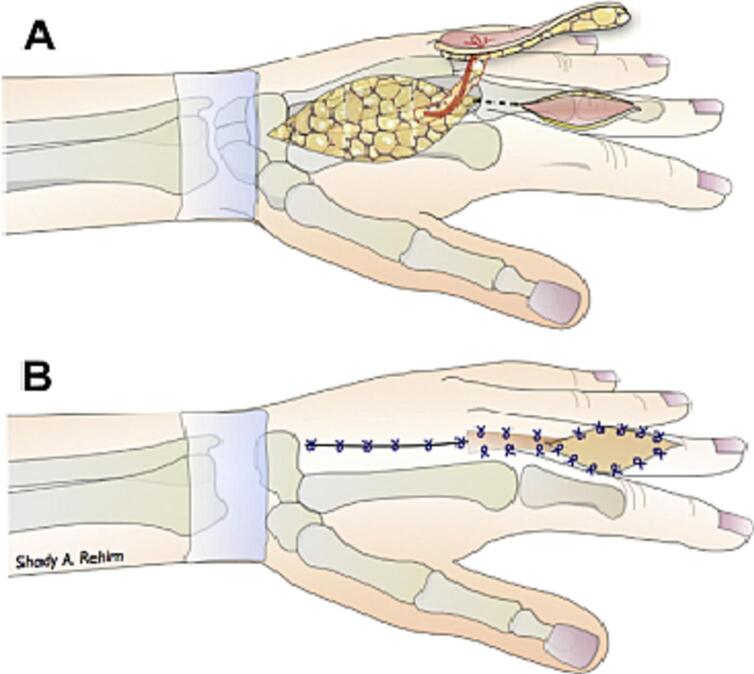
Operation technique. Elevation and inset of DMCA flap (A) to cover a defect over the dorsum of the finger (B) [3].

The patient was positioned in a prone position with their arm resting on a hand table. The surgical procedure was performed using general anesthesia, with a tourniquet applied to the upper arm. During the operation, the dorsal radial artery and its branches were identified using a doppler. The flap was created near to the proximal side of the defect and incised circumferentially (Fig. 3A). The flap was elevated as an island flap. The dissection began from the radial side of dorsal metacarpal located in the first interdigital space. An incision was made in the region between the defect and the pedicle of the flap, and subcutaneous tissue was removed to create a sulcus. The dorsoradial metacarpal flap was then reversed and passed through a skin tunnel to reach the defect site (Fig. 3B). The donor site was closed after undermining the skin flaps. After applying a non-adherent dressing, the hand was immobilized in a neutral position for approximately one week (Fig. 4).

## 4 Discussion

The reconstruction of complex soft tissue defects in the hand remains a significant challenge for plastic surgeons. Achieving successful reconstruction of hand defects is essential to enable patients to avoid postoperative hand dysfunction and facilitate a swift return to their daily activities and work [6]. Early coverage of soft tissue defects in the hands is crucial to stiffness and facilitate early return to work. An adequate coverage is necessary to replace missing skin and safeguard exposed structures [7]. In this report, there was no exposition of tendon, joint, bone or neurovascular structure that required a flap. This condition may have been readily solved with a skin graft. Skin grafts come with several disadvantages, such as a limited resemblance to the surrounding recipient site skin in terms of color match and texture, especially when meshed. They are highly susceptible to trauma, leading to potential damage. Additionally, there is a reduced sensation in the recipient site, and secondary contracture may occur. Furthermore, both the donor and recipient sites require extended wound care, prolonging the healing process [8].

The reverse dorsal metacarpal artery flap is a highly popular option for covering dorsal finger defects. There are several designs in which it can be performed, including cutaneous pedicled, subcutaneous tunnelled, and propeller flap techniques. The thin characteristic of the dorsal hand skin naturally provides an additional advantage for the RDMA flap over other choices for dorsal finger reconstruction [9].

Extensive research has been conducted on the arterial anatomy of the dorsal finger and hand. The first and second dorsal metacarpal arteries have their origin from the radial artery and the dorsal metacarpal arch, while the remaining arteries originate from the communicating branch of the palmar arteries at the metacarpal base [9]. Complications related to this type of surgery may present as venous congestion or arterial insufficiency [4]. In our case, the complication occurred in the form of venous congestion.

## 5 Conclusion

The reverse dorsal radial metacarpal artery flap is an option for reconstructing dorsal finger defects. Its early implementation can help to preserve critical structures, reduce morbidity, and facilitate early rehabilitation.

## Consent

The patient provided written informed consent for the publication of this case report and the accompanying images. A copy of the written consent is available for review by the Editor-in-Chief of this journal on request.

## Ethical approval

Ethical approval for this study (780/VI/HREC/2023) was provided by the Health Research Ethics Comitte on 10 May 2023.

## Funding

This case report did not receive any specific grant from funding agencies.

## CRediT authorship contribution statement

Dedy Chandra Hariyono: Concept and design of study, data collection, data interpretation and analysis, drafting, revision, approval of final manuscript

Affandi Wiramur: Data collection, revision, approval of final manuscript

Amru Sungkar: Data collection, revision, approval of final manuscript

Kristanto Yuli Yarso: Revision and approval of proof manuscript

Richard Philo: Design of study, revision, approval of final manuscript

## Guarantor

Dedy Chandra Hariyono, Affandi Wiramur, Amru Sungkar, Kristanto Yuli Yarso, Richard Philo

## Declaration of competing interest

None.
